# Supplementary material for: Chromothripsis during telomere crisis is independent of NHEJ, and consistent with a replicative origin
Source: Genome Res. 2019 May;29(5):737–49. doi: 10.1101/gr.240705.118 (PMC6499312; doi:10.1101/gr.240705.118)
Supplement: Supplemental Material [file supp_gr.240705.118_Supplemental_file_1.zip › contigs/annotated_contigs/DB81/contig.2.DB81_length_629_mean_cov_6.91573926868.docx]

**DB81_length_629_mean_cov_6.91573926868**

```
TTGTTGATGCACATTTGGATCATTTCATTTTTTTTCTTGGGTTAATACTTAGGAGTTGGATAGTTGTATCATAGAGTAGGTATATGTTT
 >chr13:61342126-61342423 + E=4e-159 p=2e-02
ACATTTAAAGAAACTGCCAAAATATTTTCCAAAGTGGTTATGCCACTTCACTTTTTTTTTCAAGTTGAGAAAAATTTACTCATGTGTTA

GCAAAGGATGCATAATATGAAATTAGAGCCTATTAATCTTTATTTTTTTAACTTTTAAGTTAAAGGTACAAGTTCAGGTTTGTTACATA

GGTAAACTTATGCCATGGGGGTTTGTT|GT|CAGCATTTTTAATTTTAGCTATTCTAATAGGCACAACGTAGTGTCTTGTTGTGATTTT
                            >chr13:61343061-61343396 + E=7e-187
AATCTGCATTTTACTAATGACTAATTATGCTGTTTTTGTCAAGTTTGTCGAAGATCAGATAATTGTAGATGTGTGGTATTATTTCTGAG

GGCTCTGTTCTGTTCCATTGGTCTATATCTCTGTTTTGGTACCAGTACCATGCTATTTTGGTTACTGTAGCCTTGTAGTATAGTTTGAA

GTCAGGTAGTGTGATGCCTCCAGCTTTGTTCTTTTGGCTTAGGATTATCATGGCAACGCGGGCTTTTTTTGGTTCCATATGAACATTAA

AGTGTTTT
```
